# Genetic factors associated with intestinal metaplasia in a high risk Singapore-Chinese population: a cohort study

**DOI:** 10.1186/1471-230X-9-76

**Published:** 2009-10-13

**Authors:** Feng Zhu, Marie Loh, Jeffrey Hill, Sumarlin Lee, King Xin Koh, Kin Wai Lai, Manuel Salto-Tellez, Barry Iacopetta, Khay Guan Yeoh, Richie Soong, the Singapore Gastric Cancer Consortium

**Affiliations:** 1 Department of Medicine, National University of Singapore, Singapore, Singapore; 2 Cancer Science Institute of Singapore, National University of Singapore, Singapore, Singapore; 3 Experimental Therapeutics Centre, Agency for Science, Technology and Research (A*STAR), Singapore, Singapore; 4 Department of Pathology, National University of Singapore, Singapore, Singapore; 5 School of Surgery, The University of Western Australia, Perth, Australia

## Abstract

**Background:**

Intestinal metaplasia (IM) is an important precursor lesion in the development of gastric cancer (GC). The aim of this study was to investigate genetic factors previously linked to GC risk for their possible association with IM. A total of 18 polymorphisms in 14 candidate genes were evaluated in a Singapore-Chinese population at high risk of developing GC.

**Methods:**

Genotype frequencies were compared between individuals presenting with (n = 128) or without (n = 246) IM by both univariate and multivariate analysis.

**Results:**

Carriers of the *NQO1 *609 T allele showed an association with IM in individuals who were seropositive for *Helicobacter pylori *(HP+; OR = 2.61, 95%CI: 1.18-5.80, *P *= .018). The *IL-10 *819 C allele was also associated with IM in HP+ individuals (OR = 2.32, 95%CI: 1.21-4.43, *P *= 0.011), while the *PTPN11 *A allele was associated with IM in HP- individuals (OR = 2.51, 95%CI: 1.16-5.40, *P *= 0.019), but showed an inverse association in HP+ subjects (OR = 0.46, 95%CI: 0.21-0.99, *P *= 0.048).

**Conclusion:**

Polymorphisms in *NQO1*, *IL-10 *and *PTPN11*, in combination with HP status, could be used to identify individuals who are more likely to develop IM and therefore GC.

## Background

Gastric cancer (GC) is the second leading cause of cancer-related mortality worldwide, with more than 700,000 deaths annually[1]. The late presentation of this disease is the main reason for the high mortality and highlights the importance of early detection[2]. In Japan, mass screening programs began in the 1960's and led to a significant increase in the proportion of GC diagnosed at an early stage from 8% in 1960-1964 to 50% in 1975-1979. However, more cost-effective screening programs that target high risk groups are needed because of the limited resources available in many Asian countries. Positive assessment of *Helicobacter pylori *(HP) infection can help to identify high risk individuals since this is a proven risk factor for GC[3,4]. Various genetic factors have also been associated with an increased risk for the development of GC [5-8]. These polymorphisms could be used in conjunction with HP status and together with dietary and environmental factors to target screening programs towards individuals deemed to be at high risk.

GC is thought to arise via a multi-step pathway that involves intestinal metaplasia (IM) as a precursor lesion[9]. It has been estimated that 0.25-1.1% of IM lesions will progress to GC annually, representing an 18-78-fold increased lifetime risk of developing this disease in comparison to the general population[10,11]. In the present study, we have investigated a panel of 18 polymorphisms in 14 candidate genes for their association with IM precursor lesions in a Singapore-Chinese population considered to be at increased risk of GC because of age greater than 50 years. These polymorphisms were chosen for study because previous research has shown them to be risk factors for GC. They included SNPs in genes involved in the immune response (*IL-1β*, *IL-10*, *PTPN11*) [12-14], folate metabolism (*FR-α*, *MTHFR*)[15,16], cell growth (*EGF*, *HER2*) [17-19], cell survival (*STCH*)[20], cell invasion (*MMP2*)[21] and DNA damage or repair (*NQO1*, *SULT1A1*, *TP53*, *ADPRT*) [22-26].

## Methods

### Subjects

Subjects were recruited from the Gastric Cancer Epidemiology and Molecular Genetics Program (GCEP). This project is a prospective cohort study aiming to enroll 4,000 Singapore-Chinese subjects aged more than 50 years from four major public hospitals in Singapore (National University Hospital, Tan Tock Seng Hospital, Singapore General Hospital, Changi General Hospital). It offers screening by endoscopy and systematic follow-up for a minimum of 5 years [27]. Chinese subjects older than 50 years of age who met the following criteria were eligible to enroll in the study: (i) symptoms of dyspepsia (ie. bloating, distension, nausea, stomach pain etc), (ii) family history of gastric cancer, or (iii) a medical condition that required them to undergo gastroscopy. They must also be able to attend all study visits assigned to them. Subjects who could not undergo gastroscopy, had a history of stomach cancer or surgery, had a disabling illness, or were unable to provide informed consent were ineligible for the study. Clinical information including demographics, medical history and family history were obtained. Informed consent was obtained from all subjects and the study was approved by the institutional review boards of all hospitals involved. Blood samples from 374 individual subjects collected between April 2004 and December 2006 were used for genotyping in the present study.

Three biopsies from the antrum, body and cardia were collected for histopathological examination during each endoscopic surveillance episode. IM was diagnosed from mucosal biopsies in three locations (antrum, body and cardia) for each subject and by consensus amongst three pathologists according to the updated Sydney System for the classification and grading of gastritis [28]. In cases where H. pylori was identified in biopsies, eradication therapy was administered according to standard clinical guidelines. For 339/374 (91%) individuals, the HP status was determined using the Helicoblot2.1 serology test (Genelabs Diagnostics, Singapore). In individuals where this test was not performed, the HP status was determined from histological examination of biopsies from the antrum, body and cardia, as well as from past medical history. Blood samples (8 mls) were collected into Vacutainer CPT tubes (Becton Dickinson, Franklin Lakes, NJ) and the mononuclear cells isolated and stored at -80°C prior to DNA extraction using Tri-Reagent (MRC Inc, Cincinnati, OH).

### Helicoblot2.1 serology test

This serological assay uses a Western Blot nitrocellulose strip containing electrophoretically separated proteins from a bacterial lysate of an ulcer-causing type strain of H. pylori and a recombinant antigen of H. pylori (Genelabs Diagnostics, Singapore). When incubated with diluted serum/plasma, specific antibodies to the various antigens, if present, will bind to the H. pylori antigens on the strip. These bound antibodies appear as dark bands upon reaction with goat anti-human IgG conjugated with alkaline phosphatase and a 5-bromo-4-chloro-2-indolyl-phosphate/nitroblue tetrazolium substrate solution. In order to identify the various bands present, the strip is compared with reference strips of non-reactive (negative) and reactive (positive) controls run concurrently. Determination of H. pylori seropositivity was based on criteria recommended by the kit manufacturer. They consist of (1), 116 kD (CagA) positive band present with one or more of the following bands: 89 kD (VacA), 37 kD, 35 kD, 30 kD (UreA) and 19.5 kD together, or with the current infection marker, (2) the presence of any one band at 89 kD (VacA), 37 kD or 35 kD, with or without current infection marker, or (3) the presence of both 30 kD and 19.5 kD with or without current infection marker.

### Selection of gene polymorphism panel

A systematic literature search in PubMed was carried out using the terms "gastric cancer" and "polymorphism". From a total of 78 candidate polymorphisms identified, 18 were found to be significantly associated with the risk of GC and were therefore included in the current investigation of IM.

### Genotyping

Table 1 shows the PCR primers, annealing temperatures and product sizes for 17 SNPs investigated in this study by pyroseqeuncing. The 86-bp variable number of tandem repeats (VNTR) polymorphism in *ILRN *was genotyped using PCR followed by size analysis using gel electrophoresis. The primers and PCR conditions were the same as previously reported[29]. Polymorphisms were recorded in their most commonly used notation for easy cross-referencing. For PCR, 50 ng DNA was amplified in a 25 μl reaction containing 1 × FastStart Reaction Buffer, 2 mM magnesium chloride, 10 μM deoxynucleotide mix, 500 nM each of the forward and reverse primers and 1 unit FastStart Taq Polymerase (Roche Diagnostics, Mannheim, Germany). PCR cycling comprised of 4 minutes at 95°C, followed by 35 cycles of 30 seconds at 95°C, 30 seconds at the appropriate annealing temperature and 30 seconds at 72°C, before conclusion with 7 minutes at 72°C.

**Table 1:** PCR primers and dispensation sequences for pyrosequencing of 17 SNPs evaluated for association with IM.

	PCR				Pyrosequencing	
		
Locus	Forward Primer	Reverse Primer	°C	bp	Sequencing Primer	Dispensation
IL10 -- 1082 A/G	CTCAATCAAAGGATCCCCAGAGAC	AGGCTGGATAGGAGGTCCCTTACT	60	253	ACACTACTAAGGCTTCTTTG	cgagcagta
IL10 -- 819 T/C	GGCCAATTTAATCCAAGGTTT	TCTGCACTTGCTGAAAGCTTCTTA	60	207	CCTTGTACAGGTCATGTAA	gtcgatctc
IL-1B -- 511 T/C	CATGAGATTGGCTAGGGTAACAG	GCCCTCCCTGTCTGTATTGA	60	230	CAATTGACAGAGAGCTCC	atctgagca
MMP2 -- 1306 T/C	TTTCATCTCTGGGCCATTGT	TGAAGTTCTCCCTGTGACAACC	60	265	TCCCCACCCAGCACT	gctgactct
EGF +61 A/G	GTCATCCCTGCTTTCCTGTGTG	CAGAGCAAGGCAAAGGCTTAGA	60	266	CCCAATCCAAGGGTTGT	cagactgac
PTPN11 (int1) A/G	TGGACGAATGGCAAATTG	GATCAATCCCACCTGAGACAGA	60	182	TTGTCTCTAAAGGACTGTG	tgagctcat
NQ01 C609T	AACTGCATGGAATTGGTTGACTTA	TGGTGTCTCATCCCAAATATTCT	60	191	GTGGCTTCCAAGTCTTA	cgatcgtca
STCH rs2242661	AACTCGAATCCTGGACCTGATTAG	CTGGCGTTTATAATCAAACCTGTG	65	203	GCGGAAAGAGAAAGG	gctagtact
STCH rs1882881	CTATGGAAGGCTGCGAGAAC	ACTTCCAGCTACAGGCAACATT	65	213	GAGGCTTTTTCCATCA	gcagtcgtg
STCH rs12479	CTTGAAGGACCGTGTTGATGT'	GCAAAGGTCTCGGATAACAAAAA	60	312	ATGTTTCAGCACCAT	gatagctag
STCH rs9982492	TCGTGCTTACCTTGTTCACATT	AGTATGAGCCCTGCCATGA	60	193	CCACTTGTCCTTTAAGTCC	actcgactc
SULT1A1 G638A	GCCAGATCGCCTCTGAGGT	TGGGGGACGGTGGTGTAGT	65	233	CCTGGAGTTTGTGGG	tgcgagctc
ADPRT T2285C	GATACCTAAGTCGGGGGCTTTC	ACAAGCTTTCCAGGAGATCCTAAC	65	262	TGCTCCTCCAGGCCA	cagtctgat
HER2 +17ex17 A/G	GTCCCTCCCACCCCAAACTA	CTGCCGTCGCTTGATGAG	65	145	CCCTCTGACGTCCAT	gtcagatct
TP53 C215G	TCCCAAGCAATGGATGATTTGA	AAGCCCAGACGGAAACCGTAG	60	230	CAGAGGCTGCTCCCC	tgcagtgct
FR-a A1314G	AAGTGGAGACTGAGGCCCAGA	TGACCCCTCCCCACCAAC	60	183	GTGTGGCCTGCTCAA	cgagtacga
MTHFR C677T	ACTGTCATCCCTATTGGCAGGTTA	TCGGTGCATGCCTTCACAA	60	168	GAAGGTGTCTGCGGG	cgagtacga

Pyrosequencing was performed by incubating the PCR products with 3 μl of streptavidin magnetic beads (Amersham Pharmacia Biotech, Uppsala, Sweden) and 1× binding buffer (10 mM Tris-HCl, 2 M NaCl, 1 mM EDTA, 0.1% Tween 20) and mixing for 10 minutes at 37°C. The product mix was then denatured by 5 seconds incubation in 0.2 M NaOH solution and washed in annealing buffer (20 mM Tris-acetate, 2 mM magnesium acetate) for 10 seconds. The single-stranded products were transferred to an annealing buffer containing 15 pmol of the sequencing primer (Table 1) and incubated for 2 minutes at 80°C in a Hybaid Maxi 14 hybridization oven (Thermo Electron, USA). Pyrosequencing was then performed on a PSQ96MA pyrosequencer instrument (Biotage AB, Uppsala, Sweden). Samples that failed to give a genotype result after the first analysis were repeated up to two times. The genotyping success rate varied from 85-99% for the 18 polymorphisms.

### Statistics

Univariate analyses were carried out by Pearson's chi-square or the Fisher's exact test to examine for associations between genotype distributions, IM status and clinical factors. As there were more than one polymorphism investigated in *IL10 *and *STCH*, the haplotypes were also considered in the analyses. Variables found significantly associated with IM in the univariate analyses for all cases, and HP+ and HP- subgroups were entered in respective multivariate logistic regression models. The analyses were based on the assumption of a dominant genetic model. All statistical analyses were performed using SPSS 16.0 (SPSS Inc., Chicago, IL) software at the 5% significance level. The Woolf test was used to test for homogeneity of OR between two strata. As each polymorphism was tested for association with IM independently, it was not necessary to control for the family-wise error rate. Thus, no adjustment was made for multiple testing.

## Results

The characteristics of 374 subjects evaluated in this study are shown in Table 2. A total of 128 were diagnosed with IM and 246 without IM. No significant differences between IM+ and IM- groups were apparent for sex, family history of GC (including 1^st ^degree and 2^nd ^degree relatives), alcohol consumption (at least one unit of wine, beer or liquor per week) or smoking status (at least one cigarette per day for a minimum of one year). IM+ subjects showed a significantly higher incidence of HP infection and were also older (*P *< 0.05).

**Table 2:** Characteristics of study subjects in relation to the presence of IM.

	Total (%)	IM+ (%)	IM- (%)
Subjects	374	128	246
Mean age ± SD (range)	60.5 ± 7.8	62.9 ± 7.8	59.2 ± 7.5
Age 50-59 yrs	190 (51)	48 (38)*	142 (58)*
Age 60-69 yrs	133 (36)	55 (43)	78 (32)
Age ≥70 yrs	51 (13)	25 (19)	26 (10)
Male	207 (55)	72 (56)	135 (55)
Family history of GC	66 (18)	23 (18)	43 (17)
HP infection	191 (51)	84 (66)*	107 (43)*
Drinker	66 (18)	22 (17)	46 (19)
Smoker	90 (24)	30 (23)	60 (24)
Chronic gastritis	290 (78)	115 (90)	175 (71)
Atrophy gastritis	194 (52)	97 (76)	97 (39)
Dysplasia	1 (0.3)	1 (0.8)	0

* *P *< 0.05

Genotype frequencies for the 18 polymorphisms investigated for association with IM are presented in Table 3. All polymorphisms were in Hardy-Weinberg equilibrium (P > 0.05), with the exception of *IL10 *-819T/C, *NQO1 *609C/T and *TP53 *Arg72Pro. By univariate analysis, the *NQO1 *609 T allele was the only variant in the overall cohort that was significantly associated with IM (OR = 1.82, 95%CI: 1.05-3.15, *P *= 0.032). In HP- individuals, only the *PTPN11 *rs2301756 A allele was significantly associated with IM (OR = 2.51, 95%CI: 1.16-5.40, *P *= 0.019). Three polymorphisms in HP+ individuals were associated with IM in univariate analysis: the *IL-10 *819 C allele (OR = 2.32, 95%CI: 1.21-4.43, *P *= 0.011), *NQO1 *609 T allele (OR = 2.61, 95%CI: 1.18-5.80, *P *= 0.018) and *PTPN11 *A allele (OR = 0.46, 95%CI: 0.21-0.99, *P *= 0.048). The haplotypes in *IL10 *and *STCH *were not significantly associated with IM in overall cohort, HP-, as well as HP+ groups.

**Table 3:** Distribution of genotype frequencies according to IM and HP infection status

Gene polymorphism (rs number)	Genotype	IM-	IM+	HP-		HP+	
					
				IM-	IM+	IM-	IM+
*ADPRT *Val762Ala (rs1136410)	TT	71	31	33	9	38	22
	TC	117	60	64	24	53	36
	CC	33	16	13	6	20	10
							
*EGF *+61A/G (rs4444903)	AA	22	5	13	1	9	4
	AG	103	55	54	20	49	35
	GG	110	58	50	18	60	40
							
*FR-α *1314A/G (none)	GG	164	95	74	30	90	65
	GA	74	31	43	12	31	19
	AA	6	1	5	0	1	1
							
*HER2 *Ile/Val (rs1801200)	AA	174	92	87	32	87	60
	AG	60	30	29	8	31	22
	GG	1	1	1	1	0	0
							
*IL1RN *86-bp VNTR (none)	44	212	101	112	33	100	68
	24	28	18	9	4	19	14
	34	1	2	0	2	1	0
	54	0	1	0	0	0	1
	22	2	2	0	1	2	1
							
*IL-1β *-511C/T (rs16944)	CC	64	35	33	10	31	25
	CT	119	62	63	21	56	41
	TT	48	23	20	10	28	13
							
*IL-10 *-819T/C (rs1800871)	TT	131	55	57	21	74*	**34**
	TC	78	46	39	15	39	31
	CC	22	16	17	3	5	13
							
*IL-10 *-1082A/G (rs1800896)	AA	207	100	98	37	109	63
	AG	21	14	13	3	8	11
	GG	2	0	2	0	0	0
							
*MMP2 *-1306C/T (rs243865)	CC	178	79	85	28	93	51
	CT	46	22	26	8	20	14
	TT	3	2	2	0	1	2
							
*MTHFR *667C/T (rs1801133)	CC	132	77	64	23	68	54
	CT	98	42	50	16	48	26
	TT	14	7	8	2	6	5
							
*NQO1 *609C/T (rs1800566)	CC	64*	**21**	27	10	37*	**11**
	CT	143	80	78	25	65	55
	TT	28	22	13	4	5	18
							
*TP53 *Arg72Pro (rs1042522)	CC	45	16	22	7	23	9
	CG	126	78	66	23	60	55
	GG	51	26	24	10	27	16
							
*PTPN11 *rs2301756 (rs2301756)	GG	175	85	92*	**24**	83*	**61**
	GA	58	28	26	16	32	12
	AA	4	2	0	1	4	1
							
*STCH *rs12479 (rs12479)	GG	102	58	51	20	51	38
	GA	106	39	52	12	54	27
	AA	22	15	10	8	12	7
							
*STCH *rs1882881 (rs1882881)	AA	58	34	24	13	34	21
	AC	123	58	67	15	56	43
	CC	57	31	26	13	31	18
							
*STCH *rs2242661 (rs2242661)	AA	69	31	34	12	35	19
	AG	106	46	52	13	54	33
	GG	44	26	22	13	22	13
							
*STCH *rs9982492 (rs9982492)	CC	85	45	41	15	44	30
	CT	105	39	53	12	52	27
	TT	28	18	14	9	14	9
							
*SULT1A1 *638G/A (rs9282861)	GG	221	108	112	33	109	75
	GA	16	10	6	6	10	4
	AA	1	0	1	0	0	0

* Bold type denotes significant difference in genotype frequencies

In multivariate analysis that included all cases, HP status and age were significantly associated with IM, while the *NQO1 *T allele showing borderline association (Table 4). In HP- individuals, the *PTPN11 *A allele was the only factor associated with IM. However, in HP+ individuals the factors of older age and the *NQO1 *609 T allele, *IL-10 *819 C allele and *PTPN11 *A allele were all significantly associated with IM. These results suggest that HP status is an effect modifier of the association between IM and the *PTPN11 *A allele (*P *= 0.002). As it is possible that IM+/HP- cases in this study had prior unrecorded HP infection[30], subgroup analysis on cases with a "revised HP+" status (either HP+/IM-, HP+/IM+ or HP-/IM+) was also performed. Age (OR = 2.10, 95%CI: 1.24-3.56, *P *= 0.006) and *IL-10 *-819 C allele (OR = 1.82, 95%CI: 1.07-3.08, *P *= 0.027) were the only significant variables in this subgroup.

**Table 4:** Multivariate logistic regression analysis for associations with IM.

	OR for IM (95% CI)	*P*
All cases		
HP (positive *vs *negative)	2.16 (1.35 - 3.45)	0.001
Age (>60 *vs *<60 yrs)	2.21 (1.40 - 3.49)	0.001
*NQO1 *(CT/TT *vs *CC)	1.74 (0.99 - 3.06)	0.056
		
HP- cases		
Age (>60 *vs *<60 yrs)	1.92 (0.92 - 4.00)	0.082
*PTPN11 *(GA/AA *vs *GG)	2.51 (1.16 - 5.40)	0.019
		
HP+ cases		
Age (≥60 *vs *<60 yrs)	2.19 (1.15 - 4.17)	0.017
*NQO1 *(CT/TT *vs *CC)	2.61 (1.18 - 5.80)	0.018
*IL-10 *-819 (TC/CC *vs *TT)	2.32 (1.21 - 4.43)	0.011
*PTPN11 *(GA/AA vs GG)	0.46 (0.21 - 0.99)	0.048

## Discussion

In this study, 18 polymorphisms that were previously linked to GC were investigated for possible associations with IM in a Singapore-Chinese population. The assumption was made that IM represents a precursor lesion for the development of GC and hence should have similar genetic risk factors. The cohort evaluated here was considered to be at elevated risk for GC because of the selection of individuals aged >50 years[27]. As expected, older individuals and those demonstrating seropositivity for HP showed a doubling in the frequency of IM (Table 4).

Following univariate analysis, 3 genotypes were found to be associated with IM. The *NQO1 *609 T allele was associated with IM, particularly in HP+ individuals. The *IL-10 *-819 C allele was also significantly associated with IM in HP+ cases. Interestingly, the *PTPN11 *A allele in intron 3 (rs2301756) was associated with increased incidence of IM in HP- individuals but a decreased incidence in HP+ cases. In multivariate analysis, all 3 polymorphisms remained significantly associated with IM, with the exception of the *NQO1 *609 T allele which was associated with borderline significance in the overall cohort (*P *= 0.056).

Previous data lends support to our observations. *NQO1 *(NAD(P)H: quinine oxidoreductase 1) codes for a cytosolic enzyme that protects cells from oxidative damage by preventing the generation of semiquinone free radicals and reactive oxygen species[31]. The C to T substitution at nucleotide 609 in exon 6 results in a change of amino acid from Pro to Ser at codon 187[32]. Whereas the CC homozygous wildtype genotype (Pro/Pro) has full enzymatic activity, the TT genotype (Ser/Ser) completely lacks activity. The *NQO1 *609 TT genotype has been associated with an increased risk for various tumour types including gastrointestinal and urological cancers [33-36]. An increased risk of GC in patients with a family history of upper gastrointestinal cancers was also reported for the *NQO1 *609 TT genotype in a study on Chinese subjects[22]. Our observation of increased prevalence of IM in carriers of the *NQO1 *609 T allele concurs with earlier reports on its association with various cancers and can be explained by a decreased activity for the detoxification of environmental and dietary carcinogens.

The *NQO1 *C609T polymorphism was previously associated with seropositivity to HP in a Japanese study[37], thus raising the possibility that it is an indirect risk factor for IM via association with HP infection. However, we found no association between the *NQO1 *C609T polymorphism and HP infection in the present cohort (results not shown).

Carriers of the *IL-10 *-819 C allele express higher mucosal levels of *IL-10 *(interleukin 10) mRNA and experience colonization with more virulent HP strains[38]. Similar to *NQO1 *C609T, no association was observed here between the *IL-10 *T-819C polymorphism and HP infection. The current result showing the *IL-10 *-819 C allele is associated with IM is at odds with an Italian study that reported the TT genotype was associated with increased risk of IM[29] However, two studies in Chinese and German populations found no associations between *IL-10 *T-819C and IM[38,39].

Other common polymorphisms in the *IL-1β *and *TNF*-α cytokine genes have been proposed to influence the host response to HP and therefore the risk of developing GC[13,29,38-42]. The *IL-1β *C-511T and *IL-10 *A-1082G polymorphisms were investigated in this cohort, but no significant associations were found with seropositivity to HP or with the presence of IM (Table 3). Previous studies reported the *IL-1β *-511 T allele increased the risk of IM in some[38,39], but not all populations[12]. One study found an association between the *IL-10 *A-1082G polymorphism and IM[12,43], but 3 other studies did not[12,29,39].

*PTPN11 *(protein tyrosine phosphatase, non-receptor type 11) encodes for SHP-2, a protein tyrosine phosphatase thought to play a key role in intracellular signaling elicited by growth factors and cytokines[44]. Interactions between the HP cagA protein and SHP-2 in gastric epithelial cells are believed to contribute to the development of GC[45] The *PTPN11 *AA genotype was associated with reduced risk of gastric atrophy in a Japanese population of HP seropositive individuals [14,30]. In those studies, the assessment of gastric atrophy was done with serology test (pepsinogen levels). The present results on IM in HP seropositive Singapore-Chinese support these earlier observations, although the number of AA genotype individuals (n = 6) did not allow separate evaluation of this group. The diagnosis of IM was based on histology examination. The *PTPN11 *intron 3 G/A SNP may be in linkage disequilibrium with a coding marker that influences the interaction of SHP-2 with cagA and subsequent downstream signaling. However, its association with increased frequency of IM in HP negative individuals suggests it may play a role independently of this factor.

## Conclusion

In summary, we found 3 polymorphisms associated with IM in a Singapore-Chinese population that was at high risk for GC because of older age and seropositivity for HP. The value of these SNPs in facilitating more cost-effective surveillance programs awaits further validation in large, independent cohorts.

## Competing interests

The authors declare that they have no competing interests.

## Authors' contributions

FZ participated in the design of the study and its coordination, performed the statistical analysis and drafted the manuscript. ML performed the literature review/statistical analysis and drafted the manuscript. JH, KWL, MST and KGY provided clinical and biological insights for the study. SL and KXK carried out the genotyping of the samples. BI drafted the manuscript. RS participated in its design and coordination, supervised the study and drafted the manuscript. All authors read and approved the final manuscript.

## Pre-publication history

The pre-publication history for this paper can be accessed here:

http://www.biomedcentral.com/1471-230X/9/76/prepub
